# Supplementary material for: Complexome profiling of the Chlamydomonas psb28 mutant reveals TEF5 as an early PSII assembly factor
Source: Plant Cell. 2025 Mar 17;37(6):koaf055. doi: 10.1093/plcell/koaf055 (PMC12164590; doi:10.1093/plcell/koaf055)
Supplement: koaf055_Supplementary_Data [file koaf055_supplementary_data.zip › psb28_interactive.zip!/plots/intensity/qualityPlots/peptides/PREP1_Cre01.g020918.t1.1.html]

### Cre01.g020918.t1.1

Trivial name: PREP1  
SeqCoverage [median]: 5.80  
Mass: 117.28 kDa  
  
 

| Sequence | MissedCleavage | Mass [Da] | wtIntensity | muIntensity | Score |
| --- | --- | --- | --- | --- | --- |
| AAAIEAAEKAELER | 1 | 1470.77 | 0.00 | 1901.30 | 96.59 |
| GLELSSEELTK | 0 | 1204.62 | 0.00 | 1159.77 | 94.69 |
| NSEALTVPTQVNYVAK | 0 | 1732.90 | 0.00 | 705.00 | 67.46 |
| VVAVTSPEK | 0 | 928.52 | 305.39 | 0.00 | 52.72 |
| VVQQALFPSNTYR | 0 | 1521.79 | 0.00 | 599.23 | 85.53 |
|  |  |  | 305.4 | 4365.3 |  |
